# Supporting Patients with Nontuberculous Mycobacterial Pulmonary Disease: Ensuring Best Practice in UK Healthcare Settings

**DOI:** 10.3390/pharmacy12040126

**Published:** 2024-08-21

**Authors:** Toby Capstick, Rhys Hurst, Jennie Keane, Besma Musaddaq

**Affiliations:** 1 St James’s University Hospital, Leeds LS9 7TF, UK; 2 Royal Papworth Hospital NHS Foundation Trust, Cambridge CB2 0AY, UK; rhys.hurst@nhs.net; 3 Essex Partnership University NHS Foundation Trust (EPUT), Rochford SS4 1DD, UK; jennie.keane@nhs.net; 4 Department of Radiology, Royal Free Hospital NHS Foundation Trust, London NW3 2QG, UK; besma.musaddaq@nhs.net

**Keywords:** nontuberculous mycobacteria, pulmonary disease, NTM-PD, *Mycobacterium avium* complex

## Abstract

Nontuberculous mycobacterial pulmonary disease (NTM-PD) results from opportunistic lung infections by mycobacteria other than *Mycobacterium tuberculosis* or *Mycobacterium leprae* species. Similar to many other countries, the incidence of NTM-PD in the United Kingdom (UK) is on the rise for reasons that are yet to be determined. Despite guidelines established by the American Thoracic Society (ATS), the Infectious Diseases Society of America, and the British Thoracic Society, NTM-PD diagnosis and management remain a significant clinical challenge. In this review article, we comprehensively discuss key challenges in NTM-PD diagnosis and management, focusing on the UK healthcare setting. We also propose countermeasures to overcome these challenges and improve the detection and treatment of patients with NTM-PD.

## 1. Introduction

Nontuberculous mycobacterial pulmonary disease (NTM-PD) is a group of disorders caused by bacteria belonging to the genus Mycobacterium, excluding the *Mycobacterium tuberculosis* and *M. leprae* species. Nontuberculous mycobacteria (NTM) are found in the environment, and their lipid-rich cell walls render them resistant to unfavourable conditions such as extremes of temperature and pH, as well as to antibacterial agents [1,2]. Although more than 180 species of NTM have been characterised, only a few species cause pulmonary disease. *M. avium* complex (MAC)*, M. abscessus, M. kansasii,* and *M. xenopi* are the most common causative agents of NTM-PD in the United Kingdom (UK) [3,4]. NTM are broadly divided into slow-growing mycobacteria (e.g., *M. xenopi, M. avium,* and *M. kansasii*) and rapidly growing mycobacteria (e.g., *M. abscessus* and *M. chelonae*) [1].

The estimated prevalence of NTM-PD varies substantially within and among countries [5,6]. Variations in NTM case definitions and nonconformance to disease reporting guidelines can complicate estimations of NTM-PD prevalence [3,6,7]. Nevertheless, rates of NTM-PD appear to be highest in East Asia and lower in Europe and the United States [5,6,8]. There has been an increasing trend in NTM infection and NTM-PD reported in many countries, including the UK [5,6,8]. This increase in prevalence is particularly apparent in elderly populations [6,9]. In the UK, reliable local and national NTM-PD epidemiological data are currently lacking [10,11]. However, the positivity rate of NTM cultures in England, Wales, and Northern Ireland increased eight-fold between 1995 and 2012, from 0.9 to 7.6 per 100,000 [10,12,13]. Estimates of NTM-PD prevalence in the UK in 2016 varied from 6.4 to 16 per 100,000 [11,14,15]. The reasons behind the increasing reported prevalence of NTM-PD remain unknown, although greater awareness of the disease, an ageing population, a declining incidence of tuberculosis (TB), and improvements in diagnostic tools may be contributing factors [11].

The most well-established risk factors for NTM-PD include the presence of pre-existing lung diseases (such as bronchiectasis, cystic fibrosis [CF], chronic obstructive pulmonary disease [COPD], asthma, and interstitial lung disease [ILD]), a previous history of tuberculosis, and comorbidities such as rheumatoid arthritis, diabetes, cancer, cardiovascular disease, and chronic kidney disease [16,17,18,19,20,21,22]. Treatment with corticosteroids or other immunosuppressive agents, as well as tumour necrosis factor-alpha inhibitors for rheumatoid arthritis, are also risk factors [22]. Other contributions to increased risk include environmental (e.g., atmospheric humidity, exposure to soil) [23,24], immunological (e.g., abnormalities in the interferon-gamma/interleukin-12 pathway) [25,26,27], and genetic (e.g., mutations in CF transmembrane conductance regulator [*CFTR*]) factors [28,29,30]. Furthermore, patient factors including age, alcohol consumption, smoking, and gender may also affect the occurrence of NTM-PD [31,32]. Therefore, healthcare professionals (HCPs) need to maintain a high index of suspicion for NTM-PD to prompt early investigation in susceptible patient groups in order to prevent delays in diagnosis and reduce the risk of disease progression.

## 2. Challenges of NTM-PD Diagnosis

**Figure 1:**
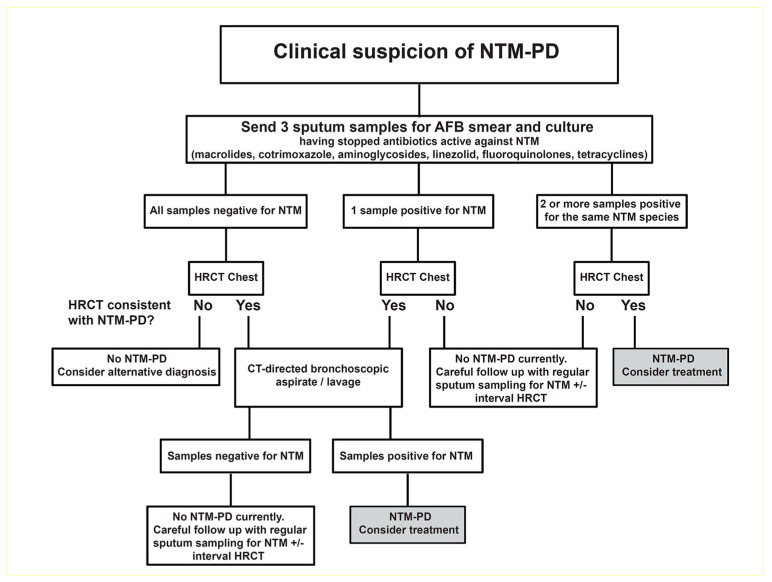
An algorithm for the investigation of suspected cases of NTM-PD. Reproduced from British Thoracic Society guidelines for the management of nontuberculous mycobacterial pulmonary disease (NTM-PD), Charles S Haworth et al., 72; iii1–ii64, 2017 with permission from BMJ Publishing Group Ltd. [12,35]. AFB = acid-fast bacilli; CT = computed tomography; HR = high-resolution; NTM = nontuberculous mycobacterial; PD = pulmonary disease.

NTM-PD diagnosis is based on clinical, microbiological, and radiological findings as described in the American Thoracic Society (ATS) and Infectious Diseases Society of America (IDSA) guidelines originally published in 2007 and in the updated international ATS/European Respiratory Society (ERS)/European Society of Clinical Microbiology and Infectious Diseases (ESCMID)/IDSA guidelines published in 2020 [33,34]. These criteria have also been adopted by the British Thoracic Society (BTS), which recommends a combination of acid-fast bacilli culture tests and computed tomography (CT) scans before considering a diagnosis of NTM-PD and the initiation of treatment (Figure 1) [12].

### 2.1. Screening Guidelines and Diagnostic Testing Procedures

The number of confirmed diagnoses of NTM-PD is likely to be underestimated due to nonadherence to NTM-PD screening guidelines [2,36]. For example, both the ERS and BTS bronchiectasis guidelines recommend testing for NTM in patients with bronchiectasis [37,38,39]. However, among patients in the UK enrolled in the EMBARC registry, testing for NTM was only performed in 17.2% of cases [37]. Geographical variations in the microbiological diagnosis of NTM-PD in the UK have also been reported, which may reflect either true differences in prevalence or that microbiological testing varies in different laboratories [10,11].

### 2.2. Clinical Awareness

Despite the recent improvements in the awareness of NTM-PD in the UK [12,15,40], further advances in the clinical awareness and understanding of NTM are warranted to improve NTM-PD diagnosis and avoid diagnostic delays. Significant efforts are required to raise awareness of NTM-PD more broadly so that HCPs working in primary care and non-respiratory specialists in secondary care consider the diagnosis in high-risk patients and those without a history of pre-existing lung disease who present with a chronic cough and non-specific constitutional symptoms. There is also a need to raise awareness of NTM-PD amongst respiratory specialists so that screening is undertaken in patients before commencing long-term treatment with macrolides (e.g., those with COPD and bronchiectasis) [10,12]. Routine screening for NTM should also be considered in other susceptible patient groups, including patients with severe disease, recurrent exacerbations, those receiving high-dose inhaled corticosteroids or other immunosuppressives, as well as those with bronchiectasis, CF, COPD, or ILD.

### 2.3. Microbiology

According to UK and international guidelines, at least three respiratory samples should be sent for mycobacterial culture in suspected NTM-PD cases [10,12]. The BTS guidelines recommend sputum induction in individuals with suspected NTM-PD who are unable to spontaneously produce sputum and when CT-directed bronchial washings are not suitable [12]. In primary care, general practitioners need to specifically request that sputum samples from patients presenting with risk factors for NTM-PD be sent for both mycobacterial culture and routine culture.

Currently, automated liquid culture technologies are the gold standard for mycobacterial culture [1,41]. The accurate identification of mycobacterial species in individuals with suspected NTM-PD is critical, as different NTM species often require treatment with different regimens [1,42]. With the rapid decrease in sequencing costs over the last few years, gene sequencing and other molecular techniques have become the method of choice for the identification of mycobacterial species. Recently, Matsumoto et al. [43] developed a novel sequencing-based approach that can identify 175 NTM species based on 7547 genomic profiles, overcoming many of the limitations of previous molecular methods for NTM identification [44]. Moreover, matrix-assisted laser desorption–ionisation–time-of-flight mass spectrometry has emerged as a promising method for NTM identification, complementing molecular analyses [45]. It cannot, however, differentiate between the three subspecies of *M. abscessus*, which is essential, as each subspecies has a different drug resistance profile [46,47].

### 2.4. Radiology

According to the BTS guidelines, CT should be performed in all individuals reporting symptoms consistent with NTM-PD, particularly in patients with bronchiectasis [12,39,48]. Ideally, supine inspiratory volumetric contiguous and high-resolution scans with a 1 mm slice thickness should be acquired [12]. Despite the wide range of NTM-PD clinical symptoms, radiological evaluation typically reveals evidence of cavitation and/or nodular–bronchiectatic disease [49,50,51]. Traditionally, the fibrocavitary phenotype of NTM-PD has been observed more commonly in men with COPD, but it is being seen increasingly in women, presumably due to the increasing incidence of COPD in women [3,51]. The cavities can be large and thick-walled, but may also be small and thin-walled, often resembling TB or malignant lesions, making it difficult to differentiate these diseases [3]. In contrast to fibrocavitary NTM-PD, nodular–bronchiectatic NTM-PD is more frequent in patients with pre-existing bronchiectasis or no history of pre-existing lung disease. Typical radiological findings of nodular–bronchiectatic NTM-PD include bronchiectasis, tree-in-bud opacity, nodules, bronchial wall thickening, and mucus plugging. This nodular–bronchiectatic form in particular can be seen in a subgroup of older women with no pre-existing medical conditions. In this cohort, the disease is often confined to the lingula and middle lobe, with MAC usually being the causative organism [3,52]. While radiological evaluation plays a critical role in the diagnosis of NTM-PD, it is also essential for monitoring disease activity in people who are not on treatment and for evaluating the radiological response during and at the end of NTM-PD treatment [53].

## 3. Challenges of NTM-PD Management

### 3.1. Decision to Treat

Predictors of NTM-PD severity and mortality may help guide clinical decision making and identify patients who are more likely to benefit from treatment. These predictors include low body mass index (BMI), malnutrition, advanced age, immunosuppression (e.g., immunosuppressive therapy, human immunodeficiency virus (HIV) infection, and primary or secondary immunodeficiencies), clinical symptoms (e.g., fever, haemoptysis, respiratory failure, weight loss), the presence of cavitation, comorbidities, and positive sputum culture and/or bronchial washing sample. Biochemical indicators of severe NTM-PD include low serum levels of albumin, low lymphocyte or erythrocyte counts, and an elevated erythrocyte sedimentation rate [54,55,56].

Comorbidities (including respiratory and cardiovascular diseases) present an increased risk for NTM-PD and are associated with worse NTM-PD outcomes; the management of such comorbidities should, therefore, be considered in treatment decisions for NTM-PD [12,22]. Given that many patients with NTM-PD may have comorbid conditions, the risk of drug interactions between the multidrug antibiotic regimens recommended to manage NTM-PD and treatments that patients may already be using for comorbid conditions must be considered. For example, macrolides are known to interact with digoxin and other antiarrhythmic drugs, as well as statins, which are used to treat cardiovascular disease [57,58]. Rifampicin-containing regimens can accelerate the metabolism of a wide variety of drugs, including corticosteroids, anticoagulants, sulphonylureas, and CFTR modulators [12]. With the prevalence of comorbidities and the likelihood of polypharmacy in patients with NTM-PD, treatment decisions can be complex. As noted, the incidence of NTM-PD is increasing most in older age groups [6,9], who are especially likely to have comorbidities requiring pharmacological intervention and are particularly challenging to manage [59]. Collaboration with pharmacists, given their knowledge of drug–drug interactions and adverse effects, can play a key role in the management of patients with NTM-PD [12,59,60]. The effects of the toxicities associated with NTM-PD treatment on any concomitant conditions should also be taken into account [12].

The clinical course of NTM-PD varies widely among patients. Despite the fact that NTM-PD can cause significant morbidity and mortality (3.6-fold increased risk of death), some patients recover without the need for treatment but may be at risk of subsequent relapse [52,61,62,63,64]. However, most patients with NTM-PD, especially those with bronchiectasis or other underlying lung diseases, require prolonged treatment (a minimum of 12 months after culture conversion) with a multidrug antibiotic regimen, which is often associated with significant toxicity [12,33,38,65]. The decision to initiate treatment takes a number of factors into account, including the mycobacterial species causing the disease, the susceptibility profile of the organism, the severity of symptoms and radiological findings, the patient’s fitness, the presence of comorbidities, and the goal of the intervention.

### 3.2. Drug Resistance

Macrolide antibiotics (e.g., clarithromycin and azithromycin) are included in current standard-of-care regimens in the treatment of *M. avium*-mediated pulmonary disease. Although relatively infrequent, macrolide resistance has been shown to limit treatment options and outcomes [12,34].

The most significant risk factor associated with the development of acquired macrolide resistance in *M. avium* is point mutations in rrl, the gene encoding 23S rRNA. Macrolide resistance is also common among NTM-PD patients infected with one of the three *M. abscessus* subspecies, as two subspecies (subsp. *abscessus* and subsp. *bolletii*) carry an inducible macrolide resistance gene (erm41) [1]. Under chronic exposure to rifamycin, *M. kansasii* may develop resistance to rifampicin-containing regimens; hence, rifamycin susceptibility should be tested in patients infected with *M. kansasii* [34].

The elimination of treatment-resistant NTM strains remains a major challenge for the treatment of refractory NTM-PD [2]. In patients infected with macrolide-resistant *M. avium*, macrolide-resistant *M. abscessus*, or rifamycin-resistant *M. kansasii*, treatment regimens should be guided by drug sensitivity testing and may include combinations of drugs such as amikacin, clofazimine, imipenem, tigecycline, co-trimoxazole, ciprofloxacin/moxifloxacin, linezolid, doxycycline/minocycline, rifabutin, isoniazid, ethambutol, and azithromycin [34]. Patients with macrolide-resistant or rifampicin-resistant NTM-PD may also benefit from surgery [12,66,67]. Other drug therapies that have been investigated include interferon-gamma and other immunotherapeutic agents, such as mammalian target of rapamycin (mTOR) inhibitors, programmed cell death-1 (PD-1)/programmed cell death ligand-1 (PD-L1) inhibitors, and haem oxygenase-1 (HO-1) inhibitors. These have not been recommended for most patients due to the limited evidence of their benefits [12,65,68,69].

Due to the lack of curative therapies, high toxicities of current treatments, and lack of accurate predictors of treatment response, deciding which patients should be treated and which treatment regimens are the most appropriate for each patient remain the most critical challenges for the management of NTM-PD [1,2,3]. These factors highlight the urgent need for more effective and safer therapies, as well as indicators of disease severity and predictors of treatment response. The lack of large-cohort randomised controlled trials is a key factor hindering the development of effective and safe therapies for NTM-PD. Most studies assessing the safety and efficacy of treatments for NTM-PD are case reports, impairing the generalisability of the findings [10]. Although guidelines from the ATS/ERS/ESCMID/IDSA and the BTS provide treatment recommendations for the most common NTM species [12,33], it is only recently that consensus recommendations have been published for the less common NTM bacteria that cause pulmonary disease [70].

## 4. Consensus on NTM-PD Management

There are national and international management guidelines for NTM-PD, and even though these guidelines are widely adopted in the UK, treatment outcomes remain unsatisfactory and response rates are low. The lack of established referral pathways from primary to secondary care and from secondary care to NTM-PD specialists along with regional variations in diagnostic testing can lead to significant delays in NTM-PD diagnosis, contributing to the poor NTM-PD treatment outcomes. Additionally, regional variations exist in the care provided to patients diagnosed with NTM-PD [10]. Despite recent evidence of person-to-person transmission of NTM in patients with CF [71,72], NTM-PD is widely believed to be a non-transmittable disease. Nevertheless, the route of infection remains unclear, impeding the establishment of effective infection control measure guidelines in this area.

The complexity of NTM-PD diagnosis and management calls for the establishment of specialised regional multidisciplinary NTM-PD centres. Currently, in the best-case scenario, patients with NTM-PD are under the care of HCPs with experience in TB, bronchiectasis, infectious diseases, or respiratory diseases (e.g., COPD). However, there is a lack of education and appropriate training in the diagnosis and management of NTM-PD even among clinicians looking after patients with COPD, evident in the fact that referrals from primary care tend to increase after education sessions. Until specialised NTM-PD multidisciplinary teams (MDTs) are established, constant education and sharing of experience among HCPs is key to minimising regional variations in the management of NTM-PD in the UK [10].

Although no treatment is provided to most NTM-PD patients with mild symptoms, long-term follow-up is still recommended for all patients [12]. The severity of respiratory symptoms, presence and severity of underlying conditions, the extent of lung damage, patient engagement, NTM pathogenicity, and drug resistance profile are all factors that determine whether treatment for NTM-PD should be initiated [10]. The treatment of NTM-PD is complex and varies based on the particular NTM species, making the accurate identification of the NTM species essential [42]. The BTS guideline includes recommendations for treating the most common NTM species causing NTM-PD: MAC, *M. kansasii*, *M. malmoense*, *M. xenopi,* and *M. abscessus* (Table 1, Table 2, Table 3, Table 4 and Table 5). Combinations of at least three antibiotic drugs are usually recommended, with a minimum duration of treatment continuing for at least 12 months after culture conversion [12]. The standard of care for patients infected with MAC, the most common cause of NTM-PD, is the combination of macrolides, rifampicin, and ethambutol given for at least 12 months after culture conversion. However, significant variations exist in the reported efficacy of this regimen. A recent meta-analysis of 16 studies involving 1462 patients with NTM-PD found that this combination provided poor treatment outcomes, with a culture conversion rate of only 60% [73]. Another study showed that almost half of the patients who initially responded to macrolide-containing regimens eventually relapsed [74]. Adult and post-pubescent children without CF, who are refractory to standard guideline-based treatment and remain culture positive for MAC after 6 months of treatment, are eligible to receive additional treatment with nebulised liposomal amikacin. This can be continued for 12 months after sputum culture conversion but should be stopped if culture conversion is not achieved after 6 months [75].

The complexity of drug regimens is a significant challenge for many patients due to overlapping drug toxicity profiles and the potential for drug–drug interactions with medications taken for other underlying comorbidities. In situations where first-line drugs cannot be used due to drug resistance, intolerance or drug–drug interactions, alternative options must be identified. A number of emerging treatments have been identified as potential options for NTM-PD including bedaquiline, clofazimine, tetracycline derivatives (e.g., omadacycline), oxazolidinones (e.g., tedizolid), combining β-lactams with β-lactamase inhibitors (e.g., imipenem/cilastatin with relebactam, ceftazidime with avibactam, or meropenem with varborbactam) [76].

Regardless of treatment, patients with NTM-PD should be provided with education on their drug regimens, dosing schedules, advice on the need for adherence and guidance on the management of side effects whilst being closely monitored by NTM-PD specialists. Regular, in-person clinical evaluations are recommended, initially every two to four weeks (depending on the patient’s needs) and extending to intervals of up to 6 months for stable patients, which can be held via telephone consultations. During clinic visits, serial sputum cultures should also be performed. Patients undergoing treatment for NTM-PD should be monitored for renal function, anticipated poor response or delayed response, potential drug interactions, and treatment-related toxicities, such as gastrointestinal disorders, rash, tinnitus, optic neuritis, and hepatotoxicity—regular therapeutic drug monitoring should only be employed in patients showing signs or symptoms of toxicity (or who are at risk of toxicity) [7]. Importantly, patients receiving aminoglycosides should undergo frequent audiograms, as ototoxicity is a frequent and irreversible side effect of macrolides and aminoglycosides, occurring in up to one-third of NTM-PD patients treated with such regimens; if ototoxicity is suspected, treatment should be discontinued [53,65,77]. Some anti-NTM-PD antibiotics can cause serious adverse effects of cardiotoxicity (QTc prolongation) and hepatotoxicity; hence, electrocardiogram and liver function tests should be performed before treatment initiation (baseline), after 2 weeks of treatment and repeated intermittently every 3-6 months [65].

Patients with treatment-refractory disease (defined as failure to culture convert after 12 months of NTM treatment), recurrence of disease (two positive mycobacterial cultures following culture conversion), drug-resistant disease prior to treatment, and those who may benefit from surgery require particularly careful and expert evaluation. The BTS guideline recommends lung resection surgery as an option for patients with NTM-PD with localised areas of severe disease [12].

Given the complexity of NTM-PD, an MDT management approach should be considered to ensure access to all specialities required for holistic NTM-PD management (Table 6) [78]. Specifically, NTM-PD MDTs should include an expert clinician (disease diagnosis, treatment, and monitoring), specialist respiratory nurses (treatment and monitoring, patient wellbeing), a respiratory physiotherapist (airway clearance and exercise), a microbiologist (sputum conversion, treatment monitoring), a radiologist (radiological diagnosis and monitoring), a pharmacist (drug interactions, side effect management, introduction of new treatments, and therapeutic drug monitoring), a dietitian (as weight loss is a risk factor of severe NTM-PD), and a psychologist (psychological support and management of mental health conditions due to chronic illness). The establishment of continuing medical education and training programmes is critical to ensure that NTM-PD MDTs and allied HCPs maintain competence to provide the best care possible. The establishment of a clear referral pathway consensus from primary to secondary care and from secondary care to NTM-PD specialists is also urgently required [10].

The establishment of specialised regional centres that can support local NTM-PD services is particularly crucial for patients with (1) drug-resistant NTM (e.g., *M. abscessus*) requiring prolonged treatment, (2) cavitary NTM-PD requiring surgical evaluation, (3) persistent or refractory NTM infection, and (4) co-infection with other bacteria, viruses, or fungi. Local and national clinical networks are also required to facilitate direct communication among experts managing diseases associated with NTM (e.g., CF and TB units, bronchiectasis clinics). Regional and national MDT meetings should also take place regularly to share experiences and establish or amend local and national guidelines according to the needs [10]. Many centres lack specialist HCPs such as nurses, pharmacists, dietitians, and physiotherapists to support patients throughout their treatment journey. A survey of the management of NTM-PD in UK-wide clinical practice showed substantial variation in practices throughout the UK. Across sites treating patients with NTM-PD, most of which were TB clinics, 68% had support available from clinical nurse specialists, 47% had support from physiotherapists, and 41% had support from pharmacists [79]. Specialist (clinical) pharmacists with knowledge and experience in NTM-PD are unfortunately not always available in the UK. Efforts are underway within the UK to increase general practice access to clinical pharmacists [80], whose knowledge can positively impact health outcomes, particularly in cases of polypharmacy and long-term conditions [10,81]. However, the availability of such specialists remains low; therefore, such expertise cannot always be made available to patients with NTM-PD. In the authors’ opinion, the formation of both local and national networks could help build awareness of NTM-PD amongst pharmacists in primary and secondary care, mitigating the need for patients to be solely managed by specialist pharmacists, through the use of referral pathways for advice and guidance and harmonising the quality of care for all patients. Additionally, pharmacists taking a role within hospital MDTs can be of great benefit, such as in-home intravenous antibiotics services to prevent unnecessarily long hospital stays, where the pharmacist can advise on appropriate intravenous drug regimens, the administration of drugs, and drug stability. Moreover, the establishment of MDTs for NTM-PD-related services can be informed from the experience gained in the successful establishment of MDTs in other areas of lung disease (e.g., CF and bronchiectasis), supporting patient access to specialist HCPs [10].

Patients can benefit from education and support after a diagnosis of NTM-PD [10], but these have been limited in the UK. The NTM Patient Care UK Association [82] was established in 2018 to improve access to information for patients with this disease. NTM Patient Care UK has recently released patient-directed materials to help with patient education [83,84]. Tools to assist patients in communicating with HCPs can also be beneficial.

To address the need for consistent, standardised, high-quality care for patients with NTM-PD, NTM Network UK, in partnership with HCPs, patients, and professional associations, launched the first Standards of Care for NTM-PD in 2024 [85].

## 5. Concluding Remarks

Further research is required to understand the differences in regional variations in NTM-PD prevalence, management, and referral pathways in the UK, as well as to develop more efficient therapeutic strategies to target different NTM species. There remain significant clinical challenges in the diagnosis and treatment of NTM-PD, and international guidelines are predominantly based on experience and case studies. The lack of effective evidence-based treatments, the development of mutational resistance, poor adherence to guidelines, drug toxicity leading to poor adherence, late diagnosis due to lack of physician awareness, and the lack of regional specialist centres and referral pathways are all factors contributing to the poor NTM-PD treatment outcomes. Mitigating these factors is paramount to improving treatment outcomes in patients with NTM-PD.

## Figures and Tables

**Table 1:** Suggested antibiotic regimens for adults with *M. avium* complex pulmonary disease [12].

*M. avium* Complex Pulmonary Disease	Antibiotic Regimen
**Non-severe MAC pulmonary disease** (i.e., AFB smear-negative respiratory tract samples, no radiological evidence of lung cavitation or severe infection, mild-moderate symptoms, no signs of systemic illness)	Rifampicin 600 mg 3× per week and Ethambutol 25 mg/kg 3× per week and Azithromycin 500 mg 3× per week or clarithromycin 1 g in two divided doses 3× per week Antibiotic treatment should continue for a minimum of 12 months after culture conversion
**Severe MAC pulmonary disease** (i.e., AFB smear-positive respiratory tract samples, radiological evidence of lung cavitation/severe infection, or severe symptoms/signs of systemic illness)	Rifampicin 600 mg daily and Ethambutol 15 mg/kg daily and Azithromycin 250 mg daily or clarithromycin 500 mg twice dailyand consider intravenous amikacin for up to 3 months or nebulised amikacin Antibiotic treatment should continue for a minimum of 12 months after culture conversion.
**Clarithromycin-resistant MAC pulmonary disease**	Rifampicin 600 mg daily and Ethambutol 15 mg/kg daily and Isoniazid 300 mg (+pyridoxine 10 mg) daily or moxifloxacin 400 mg daily and consider intravenous amikacin for up to 3 months or nebulised amikacin Antibiotic treatment should continue for a minimum of 12 months after culture conversion.

Reproduced from British Thoracic Society guidelines for the management of nontuberculous mycobacterial pulmonary disease (NTM-PD), Charles S Haworth et al., 72; iii1–ii64, 2017 with permission from BMJ Publishing Group Ltd. [12]. AFB = acid-fast bacilli; MAC = *M. avium* complex.

**Table 2:** Suggested antibiotic regimens for adults with *M. kansasii* pulmonary disease [12].

*M. kansasii* Pulmonary Disease	Antibiotic Regimen
**Rifampicin-sensitive *M. kansasii* pulmonary disease**	Rifampicin 600 mg daily and Ethambutol 15 mg/kg daily and Isoniazid 300 mg (with pyridoxine 10 mg) daily or azithromycin 250 mg daily or clarithromycin 500 mg twice daily Antibiotic treatment should continue for a minimum of 12 months after culture conversion.

Reproduced from British Thoracic Society guidelines for the management of nontuberculous mycobacterial pulmonary disease (NTM-PD), Charles S Haworth et al., 72; iii1–ii64, 2017 with permission from BMJ Publishing Group Ltd. [12].

**Table 3:** Suggested antibiotic regimens for adults with *M. malmoense* pulmonary disease [12].

*M. malmoense* Pulmonary Disease	Antibiotic Regimen
**Non-severe *M. malmoense*-pulmonary disease** (i.e., AFB smear-negative respiratory tract samples, no radiological evidence of lung cavitation or severe infection, mild-moderate symptoms, no signs of systemic illness)	Rifampicin 600 mg daily and Ethambutol 15 mg/kg daily and Azithromycin 250 mg daily or clarithromycin 500 mg twice daily Antibiotic treatment should continue for a minimum of 12 months after culture conversion.
**Severe *M. malmoense* pulmonary disease** (i.e., AFB smear-positive respiratory tract samples, radiological evidence of lung cavitation/severe infection or severe symptoms/signs of systemic illness)	Rifampicin 600 mg daily and Ethambutol 15 mg/kg daily and Azithromycin 250 mg daily or clarithromycin 500 mg twice daily and consider intravenous amikacin for up to 3 months or nebulised amikacin Antibiotic treatment should continue for a minimum of 12 months after culture conversion.

Reproduced from British Thoracic Society guidelines for the management of nontuberculous mycobacterial pulmonary disease (NTM-PD), Charles S Haworth et al., 72; iii1–ii64, 2017 with permission from BMJ Publishing Group Ltd. [12]. AFB = acid-fast bacilli.

**Table 4:** Suggested antibiotic regimens for adults with *M. xenopi* pulmonary disease [12].

*M. xenopi* Pulmonary Disease	Antibiotic Regimen
**Non-severe *M. xenopi* pulmonary disease** (i.e., AFB smear-negative respiratory tract samples, no radiological evidence of lung cavitation or severe infection, mild-moderate symptoms, no signs of systemic illness)	Rifampicin 600 mg daily and Ethambutol 15 mg/kg daily and Azithromycin 250 mg daily or clarithromycin 500 mg twice daily and Moxifloxacin 400 mg daily or isoniazid 300 mg (+pyridoxine 10 mg) daily Antibiotic treatment should continue for a minimum of 12 months after culture conversion.
**Severe *M. xenopi* pulmonary disease** (i.e., AFB smear-positive respiratory tract samples, radiological evidence of lung cavitation/severe infection, or severe symptoms/signs of systemic illness)	Rifampicin 600 mg daily and Ethambutol 15 mg/kg daily and Azithromycin 250 mg daily or clarithromycin 500 mg twice daily and Moxifloxacin 400 mg daily or isoniazid 300 mg (+pyridoxine 10 mg) daily and consider intravenous amikacin for up to 3 months or nebulised amikacin Antibiotic treatment should continue for a minimum of 12 months after culture conversion.

Reproduced from British Thoracic Society guidelines for the management of nontuberculous mycobacterial pulmonary disease (NTM-PD), Charles S Haworth et al., 72; iii1–ii64, 2017 with permission from BMJ Publishing Group Ltd. [12]. AFB = acid-fast bacilli.

**Table 5:** Suggested antibiotic regimens for adults with *M. abscessus* pulmonary disease [12].

*M. abscessus*	Antibiotic Regimen
**Clarithromycin-sensitive isolates or inducible macrolide-resistant isolates**	**Initial phase: ≥1 month ^†^** Intravenous amikacin 15 mg/kg daily or 3× per week ^‡^ and Intravenous tigecycline 50 mg twice daily and where tolerated Intravenous imipenem 1 g twice daily and where tolerated Oral clarithromycin 500 mg twice daily or oral azithromycin 250–500 mg daily **Continuation phase:** Nebulised amikacin ^‡^ and Oral clarithromycin 500 mg twice daily or azithromycin 250–500 mg daily and 1–3 of the following antibiotics guided by drug susceptibility results and patient tolerance: Oral clofazimine 50–100 mg daily ^§^ Oral linezolid 600 mg daily or twice daily Oral minocycline 100 mg twice daily Oral moxifloxacin 400 mg daily Oral co-trimoxazole 960 mg twice daily
**Constitutive macrolide-resistant isolates**	**Initial phase: ≥1 month ^†^** Intravenous amikacin 15 mg/kg daily or 3× per week ^‡^ and Intravenous tigecycline 50 mg twice daily and where tolerated intravenous imipenem 1 g twice daily **Continuation phase:** Nebulised amikacin ^‡^ and 2–4 of the following antibiotics guided by drug susceptibility results and patient tolerance: Oral clofazimine 50–100 mg daily ^§^ Oral linezolid 600 mg daily or twice daily Oral minocycline 100 mg twice daily Oral moxifloxacin 400 mg daily Oral co-trimoxazole 960 mg twice daily

^†^ Due to the poorer response rates in patients with inducible or constitutive macrolide-resistant isolates and the greater efficacy of antibiotics administered through the intravenous route, extending the duration of intravenous antibiotic therapy to 3–6 months in those that can tolerate it may be the most appropriate treatment strategy in this subgroup of patients. ^‡^ Substitute intravenous/nebulised amikacin with an alternative antibiotic if the *M. abscessus* is resistant to amikacin (i.e., minimum inhibitory concentration >64 mg/L or known to have a 16S ribosomal ribonucleic acid gene mutation conferring constitutive amikacin resistance).^§^ Start clofazimine during the initial phase of treatment if tolerated as steady-state serum concentrations may not be reached until ≥30 days of treatment. Reproduced from British Thoracic Society guidelines for the management of nontuberculous mycobacterial pulmonary disease (NTM-PD), Charles S Haworth et al., 72; iii1–ii64, 2017 with permission from BMJ Publishing Group Ltd. [12].

**Table 6:** Roles of the different MDT stakeholders in NTM-PD diagnosis and management.

MDT Member	Stage of Patient Journey	
	Screening and Diagnosis	Treatment
Clinician	Managing patient referrals. Organising imaging and laboratory testing during screening and diagnosis.	Leading the NTM-PD service. Reviewing findings from imaging or laboratory testing and ordering additional tests/scans where applicable to monitor patient progress and symptoms. Making decisions, in collaboration with other MDT members and the patient. Liaising with primary/secondary care colleagues as well as keeping patients and carers informed. Monitoring patient outcomes (e.g., databases for recording information).
		
Nurse	Reviewing patient’s clinical records in both primary and secondary care with respect to microbiology requests. If sample is requested via GP and only one sample is sent, nurse would contact GP and recommend requesting further samples and suggest referral to lead respiratory consultant for TB/NTM-PD. If requested from secondary care, nurse would discuss with clinician requesting and organise/arrange further sputum samples and CT scan if necessary and request referral to lead in TB/NTM baseline prior to treatment; ECG, visual acuity, Ishihara. Education, care plan, follow-up, sputum, ECG, visual acuity, Ishihara, drug monitoring levels, FBCs, U/Es tests, CRP tests, HIV tests.	Providing ongoing support, face-to-face clinics, home visits, telephone contact, and more recently, virtual clinic. Regular weights, sputum reviews, optic neuritis check, repeat eye testing, refer to ophthalmology. If necessary, repeating blood tests, LFT, U/E tests; drug level monitoring; and arranging/coordinating chest X-rays/CT scans. Arranging/coordinating repeat prescriptions. Reporting issues with clinical progress and side effects outside the scope of their clinical practice. Discussing patient’s progress in monthly MDT meetings. Arranging audiology testing if nebulised amikacin is required and organising nebulised amikacin trial with respiratory nurse specialist/physiotherapist. May require referral to other specialists, e.g., dietitian, physiotherapy, specialist NTM-PD centre, BTS multidrug resistance advice. Nurse to arrange, coordinate, and share clinical records and results to enable shared care. Ongoing education advocacy.
Radiologist	Aiding with diagnosis of NTM-PD, assessing imaging subtype, and raising the possibility of a diagnosis of NTM-PD in patients where it is not clinically suspected but imaging features are suggestive of it. In addition, the radiologist can look for ancillary findings on imaging that may give an idea of the predisposing factors.	Quantifying disease extent, monitoring disease progression and response, and looking for potential complications.
Respiratory physiotherapist	Optimising treatment of underlying conditions and supporting screening by considering possible diagnosis in high-risk patients. Possible involvement in sputum collection and/or sputum induction.	Providing airway clearance techniques in patients with long-term respiratory conditions. Key objectives of physiotherapy in patients with NTM-PD include assessment of all patients with a chronic productive cough or those who have difficulty clearing sputum; improvement of aerobic capacity and exercise tolerance. Providing advice to patients with impaired exercise capacity on participation in regular activity, including a pulmonary rehabilitation programme where appropriate. All interventions should be tailored to the patient’s symptoms, physical capability, and disease characteristics. Physiotherapy plays a key role in reducing exacerbation of symptoms such as coughing and breathlessness. This can significantly improve quality of life. Physiotherapists provide advice on the management of secondary symptoms such as breathlessness, incontinence, and performing nebulised drug reaction assessments to assess for use of mucus thinners and antibiotics. Physiotherapists focus on education to support self-management of a long-term condition/therapy and signposting resources for patients, along with acting as a service user advocate.
Dietitian		Performing a nutritional assessment and optimising nutritional status in line with the BTS guideline.
Pharmacist		Advice and recommendation of drug regimens initially and alternatives in cases of drug resistance and toxicity. Identifying potential issues with polypharmacy and drug–drug interactions. Patient education on drug regimen, dose and frequency, adherence, coping with and managing side effects. Advising healthcare professionals on recognising and managing side effects. Therapeutic drug monitoring (when and why to measure levels) and interpreting drug levels. Long-term patient follow-up clinics (face-to-face and/or virtual), focusing on medicines use, adherence, therapeutic drug monitoring and identification and management of associated side effects and drug–drug interactions. Medicines supply (supply to patients and managing short- or long-term drug shortages). Introduction of new medicines into local formularies and practice.
Microbiologist	Species identification, sputum conversion.	Treatment monitoring.
BTS = British Thoracic Society; CT = computed tomography; CRP = C-reactive protein; ECG = electrocardiogram; FBC = full blood count; GP = general practitioner; HIV = human immunodeficiency virus; LFT= liver function test; MDT = multidisciplinary team; nontuberculous mycobacterial pulmonary disease; TB = tuberculosis; U/E= urea and electrolytes.		

## Data Availability

No new data were created or analysed in this study. Data sharing is not applicable to this article.
